# Use of *Lactobacillus plantarum* Strains as a Bio-Control Strategy against Food-Borne Pathogenic Microorganisms

**DOI:** 10.3389/fmicb.2016.00464

**Published:** 2016-04-13

**Authors:** Mattia Pia Arena, Amandine Silvain, Giovanni Normanno, Francesco Grieco, Djamel Drider, Giuseppe Spano, Daniela Fiocco

**Affiliations:** ^1^Department of Science of Agriculture, Food and Environment, University of FoggiaFoggia, Italy; ^2^Laboratoire Régional de Recherche en Agroalimentaire et Biotechnologies, Institut Charles Viollette-Université Lille 1, Université de LilleLille, France; ^3^Institute of Sciences of Food Production (ISPA), Lecce Section, Consiglio Nazionale delle RicercheLecce, Italy; ^4^Department of Clinical and Experimental Medicine, University of FoggiaFoggia, Italy

**Keywords:** *Lactobacillus plantarum*, antimicrobial compounds, inhibiting activity, cell-free supernatant (CFS), organic acid, pathogens

## Abstract

*Lactobacillus plantarum* is one of the most versatile species extensively used in the food industry both as microbial starters and probiotic microorganisms. Several *L. plantarum* strains have been shown to produce different antimicrobial compounds such as organic acids, hydrogen peroxide, diacetyl, and also bacteriocins and antimicrobial peptides, both denoted by a variable spectrum of action. In recent decades, the selection of microbial molecules and/or bacterial strains able to produce antagonistic molecules to be used as antimicrobials and preservatives has been attracting scientific interest, in order to eliminate or reduce chemical additives, because of the growing attention of consumers for healthy and natural food products. The aim of this work was to investigate the antimicrobial activity of several food-isolated *L. plantarum* strains, analyzed against the pathogenic bacteria *Listeria monocytogenes, Salmonella Enteritidis, Escherichia coli* O157:H7 and *Staphylococcus aureus*. Antagonistic activity was assayed by agar spot test and revealed that strain *L. plantarum* 105 had the strongest ability to contrast the growth of *L. monocytogenes*, while strains *L. plantarum* 106 and 107 were the most active microorganisms against *E. coli* O157:H7. The antimicrobial ability was also screened by well diffusion assay and broth micro-dilution method using cell-free supernatants (CFS) from each *Lactobacillus* strain. Moreover, the chemical nature of the molecules released in the CFS, and possibly underlying the antagonistic activity, was preliminary characterized by exposure to different constraints such as pH neutralization, heating, catalase, and proteinase treatments. Our data suggest that the ability of *L. plantarum* cultures to contrast pathogens growth *in vitro* depends, at least in part, on a pH-lowering effect of supernatants and/or on the presence of organic acids. Cluster analysis was performed in order to group *L. plantarum* strains according to their antimicrobial effect. This study emphasizes the tempting use of the tested *L. plantarum* strains and/or their CFS as antimicrobial agents against food-borne pathogens.

## Introduction

Lactobacilli are widespread microorganisms which are extensively used in the food field both as technological starters in the fermented products and as probiotics due to their strain-specific healthy properties (Cebeci and Gürakan, 2003; Georgieva et al., 2009; Altay et al., 2013). Among Lactobacilli, *Lactobacillus plantarum* is one of the most versatile species, including strains with valuable technological skills and recognized probiotic features (da Silva Sabo et al., 2014; Guidone et al., 2014). Moreover, a number of probiotic *L. plantarum* strains hold multipurpose features as they can both carry out appreciable fermentative and metabolic processes, e.g., increasing the amount of specific beneficial compounds such as vitamins in the fermented food product, and promote the maintenance of consumers' health, since their capacity to modulate the host immune response and to *de novo* produce vitamins in the human gut (Arena et al., 2014, 2015). Concurrently, the increasing attention of consumers for healthy and natural food prompts food industry and scientific research to investigate the application of natural compounds for the processing of food products, in order to eliminate or reduce chemical additives used as antimicrobial agents. Thus, in recent decades, several lines of research have tried to find “the natural solution” to “the chemical problem.” Among these, the selection of microbial molecules, and/or bacterial strains able to produce such compounds, to be used as antimicrobials and preservatives, proved that Lactic Acid Bacteria (LAB) could be suitable candidates for such “natural purpose” (Šušković et al., 2010; da Silva Sabo et al., 2014).

LABs, including several *L. plantarum* strains, have been shown to produce different antimicrobial agents such as organic acids, hydrogen peroxide, diacetyl, bacteriocins, and antimicrobial peptides, with a variable spectrum of action (Herreros et al., 2005; Tharmaraj and Shah, 2009; Cortés-Zavaleta et al., 2014). Several lactobacilli, including *L. plantarum*, exhibit antagonistic activity against pathogenic and spoilage microorganisms. Such antimicrobial effect has been often ascribed to the production of organic acids, including lactic and phenyllactic acids (Tharmaraj and Shah, 2009; Neal-McKinney et al., 2012; Tejero-Sariñena et al., 2012; Rodríguez-Pazo et al., 2013). However, also the synthesis of bacteriocins and/or bacteriocin like substances has been reported to account for the antagonistic activity exerted by probiotic lactobacilli (Kos et al., 2011; Al Kassaa et al., 2014).

The antagonistic activity of selected microorganisms and/or their extracellular antibacterial agents included in the cell free supernatants (CFS) offer valuable opportunities for food preservation (Kecerová et al., 2004) as well as feed supplements or in veterinary medicine (Nousiainen et al., 2004; Bilkova et al., 2011; Cortés-Zavaleta et al., 2014). Because of their widespread association with foods and their generally recognized as safe (GRAS) status, the use of LAB and/or their metabolites as natural drugs has attracted considerable interest in recent years (Reis et al., 2012). In the food industry, the use of the bacteriocins nisin and pediocin has allowed to reduce the addition of chemical preservatives and the intense thermal treatments, thus enhancing sensory and nutritional properties without impairing safety (De Vuyst and Leroy, 2007; Sobrino-Lopez and Martin-Belloso, 2008). Moreover, several other antimicrobial peptides produced by probiotic LABs have been characterized and suggested for potential and relevant applications in food preservation and safety (Reis et al., 2012; Gupta and Srivastava, 2014).

As the antimicrobial activity of LAB bacteriocins is usually restricted to Gram-positive bacteria, organic acids and organic acid-producing LAB could have even wider applications in food safety (De Vuyst and Leroy, 2007; Mu et al., 2012). In this regard, the use of probiotics which produce antimicrobial metabolites, including organic acids, has been proposed as part of effective bio-control strategies to contrast the contamination of animal feed by spoilage and pathogenic microorganisms, and to reduce pathogen loads in livestock (Gerbaldo et al., 2012; Neal-McKinney et al., 2012), consequently decreasing food-borne illness in humans. Recently, culture supernatants from probiotic LAB, with *in vitro* inhibitory action on *Clostridium difficile* (CD), were suggested as a basis for alternative therapies to treat CD infections in humans (Joong-Su et al., 2013). Accordingly, cell-free probiotic extracts were proposed as alternative ingredients to probiotic live cells for nutritional and medicinal applications (Saadatzadeh et al., 2013).

Our main objective was to understand whether *Lactobacillus* spp. could represent a natural alternative to the chemical antimicrobials commonly used in the food preparation. Therefore, this study evaluated the antimicrobial activity of 79 wine-derived *L. plantarum* strains against seven pathogenic bacteria, generally involved in foodborne poisoning and infections. The pathogens used in this work were *Listeria monocytogenes*, which can cause abortions and/or gastrointestinal diseases leading to death (Sip et al., 2012), *Escherichia coli* O157:H7, which provokes haemorrhagic colitis and haemolytic uremic syndrome (Mead and Griffin, 1998), *Salmonella Enteritidis*, which determines abdominal pain, nausea, vomiting, and diarrhea (Liu et al., 2011), and methicillin-resistant and methicillin-sensitive strains of *Staphylococcus aureus*, which is involved in harmful gastroenteritis (Gutiérrez-Larraínzar et al., 2012). Additionally, we investigated on the chemical nature of the molecules possibly accounting for the observed antimicrobial activity.

## Materials and methods

### Bacterial strains and growth conditions

This study was carried out on 79 *L. plantarum* strains deposited into the culture collection of Foggia University (Italy; UNIFG) and previously isolated from wine and must (Table 1). All *L. plantarum* strains were growth on de Man–Rogosa–Sharpe (MRS; Sigma-Aldrich, St. Louis, MO, USA) at 30°C.

**Table 1:** *****Lactobacillus plantarum*** strains used in this work**.

	**No. collection**	**Name**	**Isolation source**		**No. collection**	**Name**	**Isolation source**
1	UNIFG 6	*Lactobacillus plantarum*	wine	41	UNIFG 74	*Lactobacillus plantarum*	wine
2	UNIFG 9	*Lactobacillus plantarum*	wine	42	UNIFG 75	*Lactobacillus plantarum*	wine
3	UNIFG 10	*Lactobacillus plantarum*	wine	43	UNIFG 79	*Lactobacillus plantarum*	wine
4	UNIFG 22	*Lactobacillus plantarum*	wine	44	UNIFG 80	*Lactobacillus plantarum*	wine
5	UNIFG 30	*Lactobacillus plantarum*	wine	45	UNIFG 81	*Lactobacillus plantarum*	wine
6	UNIFG 31	*Lactobacillus plantarum*	wine	46	UNIFG 82	*Lactobacillus plantarum*	wine
7	UNIFG 32	*Lactobacillus plantarum*	wine	47	UNIFG 83	*Lactobacillus plantarum*	wine
8	UNIFG 33	*Lactobacillus plantarum*	wine	48	UNIFG 84	*Lactobacillus plantarum*	wine
9	UNIFG 35	*Lactobacillus plantarum*	wine	49	UNIFG 85	*Lactobacillus plantarum*	wine
10	UNIFG 36	*Lactobacillus plantarum*	wine	50	UNIFG 86	*Lactobacillus plantarum*	wine
11	UNIFG 37	*Lactobacillus plantarum*	wine	51	UNIFG 87	*Lactobacillus plantarum*	must
12	UNIFG 38	*Lactobacillus plantarum*	wine	52	UNIFG 88	*Lactobacillus plantarum*	wine
13	UNIFG 44	*Lactobacillus plantarum*	must	53	UNIFG 89	*Lactobacillus plantarum*	must
14	UNIFG 45	*Lactobacillus plantarum*	wine	54	UNIFG 90	*Lactobacillus plantarum*	must
15	UNIFG 46	*Lactobacillus plantarum*	wine	55	UNIFG 91	*Lactobacillus plantarum*	must
16	UNIFG 47	*Lactobacillus plantarum*	must	56	UNIFG 92	*Lactobacillus plantarum*	wine
17	UNIFG 48	*Lactobacillus plantarum*	must	57	UNIFG 93	*Lactobacillus plantarum*	wine
18	UNIFG 49	*Lactobacillus plantarum*	wine	58	UNIFG 94	*Lactobacillus plantarum*	must
19	UNIFG 50	*Lactobacillus plantarum*	must	59	UNIFG 95	*Lactobacillus plantarum*	must
20	UNIFG 51	*Lactobacillus plantarum*	wine	60	UNIFG 96	*Lactobacillus plantarum*	must
21	UNIFG 52	*Lactobacillus plantarum*	must	61	UNIFG 97	*Lactobacillus plantarum*	wine
22	UNIFG 53	*Lactobacillus plantarum*	wine	62	UNIFG 98	*Lactobacillus plantarum*	wine
23	UNIFG 54	*Lactobacillus plantarum*	must	63	UNIFG 99	*Lactobacillus plantarum*	wine
24	UNIFG 55	*Lactobacillus plantarum*	wine	65	UNIFG 103	*Lactobacillus plantarum*	wine
25	UNIFG 56	*Lactobacillus plantarum*	wine	66	UNIFG 104	*Lactobacillus plantarum*	wine
26	UNIFG 57	*Lactobacillus plantarum*	wine	67	UNIFG 105	*Lactobacillus plantarum*	wine
27	UNIFG 58	*Lactobacillus plantarum*	wine	68	UNIFG 106	*Lactobacillus plantarum*	wine
28	UNIFG 59	*Lactobacillus plantarum*	must	69	UNIFG 107	*Lactobacillus plantarum*	wine
29	UNIFG 60	*Lactobacillus plantarum*	must	70	UNIFG 108	*Lactobacillus plantarum*	wine
30	UNIFG 61	*Lactobacillus plantarum*	must	71	UNIFG 109	*Lactobacillus plantarum*	wine
31	UNIFG 62	*Lactobacillus plantarum*	must	72	UNIFG 115	*Lactobacillus plantarum*	wine
32	UNIFG 63	*Lactobacillus plantarum*	wine	73	UNIFG 117	*Lactobacillus plantarum*	wine
33	UNIFG 66	*Lactobacillus plantarum*	wine	74	UNIFG 118	*Lactobacillus plantarum*	wine
34	UNIFG 67	*Lactobacillus plantarum*	wine	75	UNIFG 119	*Lactobacillus plantarum*	wine
35	UNIFG 68	*Lactobacillus plantarum*	wine	76	UNIFG 120	*Lactobacillus plantarum*	wine
36	UNIFG 69	*Lactobacillus plantarum*	wine	77	UNIFG 121	*Lactobacillus plantarum*	wine
37	UNIFG 70	*Lactobacillus plantarum*	must	78	UNIFG 122	*Lactobacillus plantarum*	wine
38	UNIFG 71	*Lactobacillus plantarum*	wine	79	UNIFG 134	*Lactobacillus plantarum*	wine
39	UNIFG 72	*Lactobacillus plantarum*	wine				
40	UNIFG 73	*Lactobacillus plantarum*	must				

The pathogenic bacteria used were: *L. monocytogenes* CECT 4032; *S. Enteritidis* CECT 409, *E. coli* O157:H7 CECT 4267, two methicillin-resistant strains of *S. aureus* MSSA1220, and *S. aureus* MRSA1209, two methicillin-susceptible strains of *S. aureus* MRSA1208 and *S. aureus* MRSA1070. All pathogens were grown in tryptone soy broth (TBS, Oxoid) and incubated at 37°C, with the exception of *S. aureus* strains that were grown in Brain Heart Infusion broth (BHI, Oxoid).

### Antimicrobial activity

The antimicrobial activity was evaluated by (i) agar spot test, (ii) well-diffusion method, and (iii) broth microdilution method. The agar spot test was carried out according to Gaudana et al. (2010). Briefly, overnight cultures of lactobacilli were spotted (5 μL) on MRS agar and incubated for 24 h at 37°C. Pathogen overnight cultures were mixed 1:100 with TSB or BHI soft agar (containing 0.6% agar, w/v) and poured over MRS agar plates containing the developed colonies of lactobacilli. Plates were incubated for 24 h and the radii of the inhibition zones were measured.

For the well diffusion assays, cultures of lactobacilli were grown in MRS broth (pH 6.5) for 18 h and, then, centrifuged (8000 × g for 20 min, 4°C). The cell-free supernatant (CFS) was recovered and sterilized by filtration through Millex-GV 0.22 μm hydrophilic Durapore PVDF membrane (Millipore, Billerica, MA, USA). To investigate on the chemical nature of the potentially inhibitory substances secreted by each *L. plantarum* strain, showing antagonistic effects, filtered CFSs (CFS-A) were submitted to different treatments. An aliquot of filtered CFS was sequentially treated according to Herreros et al. (2005). First of all, CFSs (CFS-A) were heated at 80°C for 10 min (CFS-B) and neutralized with 2 M NaOH (to pH 6.5; CFS-C), in order to rule out inhibiting effects due to organic acids. The neutralized CFSs (CFS-C) were subjected to the following treatments: (i) catalase digestion (1 mg/ml; Sigma-Aldrich Corporation, USA) at 37°C for 1 h, in order to eliminate the possible inhibitory action of hydrogen peroxide (CFS-D); (ii) separate digestion at 37°C for 2 h with different proteases, i.e., proteinase K (CFS-E; 1 mg/ml), trypsin (CFS-F, 1 mg/ml), α-chemotrypsin (CFS-G, 1 mg/ml), and papain (CFS-H, 1 mg/ml; all purchased from from Sigma, USA); (iii) heating at 80°C for 60 (CFS-I) and 90 min (CFS-L), 100°C for 60 (CFS-M) and 90 min (CFS-N), and at 121°C for 15 min (CFS-O).

Another aliquot of filtered CFS was separately treated according to Cortés-Zavaleta et al. (2014). A part of this aliquot (CFS-A^*^) was heated at 80°C for 10 min (CFS-B^*^) and then neutralized with 2 M NaOH (to pH 6.5) (CFS-C^*^), as mentioned above. Another portion of CFS-A^*^ was exposed to catalase (1 mg/ml; 37°C for 1 h; CFS-D^*^), or to different proteases, i.e., proteinase K (CFS-E^*^; 1 mg/ml, 37°C for 2 h), trypsin (CFS-F^*^, 1 mg/ml, 37°C for 2 h), α-chemotrypsin (CFS-G^*^, 1 mg/ml, 37°C for 2 h), and papain (CFS-H^*^, 1 mg/ml, 37°C for 2 h), or to different thermal treatments, i.e., 80°C for 60 (CFS-I^*^) and 90 min (CFS-L^*^), 100°C for 60 (CFS-M^*^) and 90 min (CFS-N^*^), and 121°C for 15 min (CFS-O^*^).

All treated CFSs were collected and 100 μl of each were used to fill 6 mm diameter wells previously punched on MRS agar plates. The plates were incubated for 2 h at 4°C in order to permit CFSs diffusion onto MRS agar. Overnight cultures of pathogenic bacteria were inoculated (1% v/v) into fresh TSB or BHI soft agar (0.6% agar, w/v) and poured over MRS agar plate containing CFSs. All plates were incubated at 37°C for 24 h and, then, the inhibition zones around the wells were measured.

Broth microdilution assays were assessed as described by Mayrhofer et al. (2008). Overnight cultures of pathogenic bacteria were inoculated (1% v/v) into fresh medium, i.e., TSB or BHI, and seeded in 96-well plates (Costar-Corning Incorporated, Corning, NY, USA). 200 μl of test solution consisting of 100 μl of pathogenic culture and 100 μl of CFS were mixed into the wells. Untreated CFSs (CFS-A) were diluted in physiological solution (NaCl 8.5 g/L) and used at different percentages, i.e., 5, 10, 15, 20, 25, 30, 35, 40, 45, 50% in the final volume (200 μl), in order to determine the minimum percentage of CFS able to inhibit the growth of target pathogens. Plates were incubated at 37°C for 24 h and growth of pathogenic bacteria was monitored by measuring optical density (OD_600_ nm). Furthermore, after identifying the minimum inhibiting percentage, treated CFSs were also used against pathogenic bacteria. The antimicrobial activity was expressed as inhibition (%) of pathogen growth relative to the control (i.e., pathogen grown in optimal conditions, without CFS).

### Lyophilization of cell-free supernatant

The supernatants of lactobacilli were collected by centrifugation and 10-fold concentrated by lyophilization as reported by Bermudez-Brito et al. (2013).

### Statistical analysis

Three independent experiments were conducted for all trials. Cluster analysis was used to determine the grouping of lactobacilli according to their antimicrobial activity against target pathogens. Statistical comparisons were performed by one-way ANOVA test (*p* < 0.005 was considered as statistically significant). All statistical study was performed using IBM SPSS Statistics 21.0 software program (IBM, Armonk, NY, USA).

## Results

### Agar spot test

**Figure 1:**
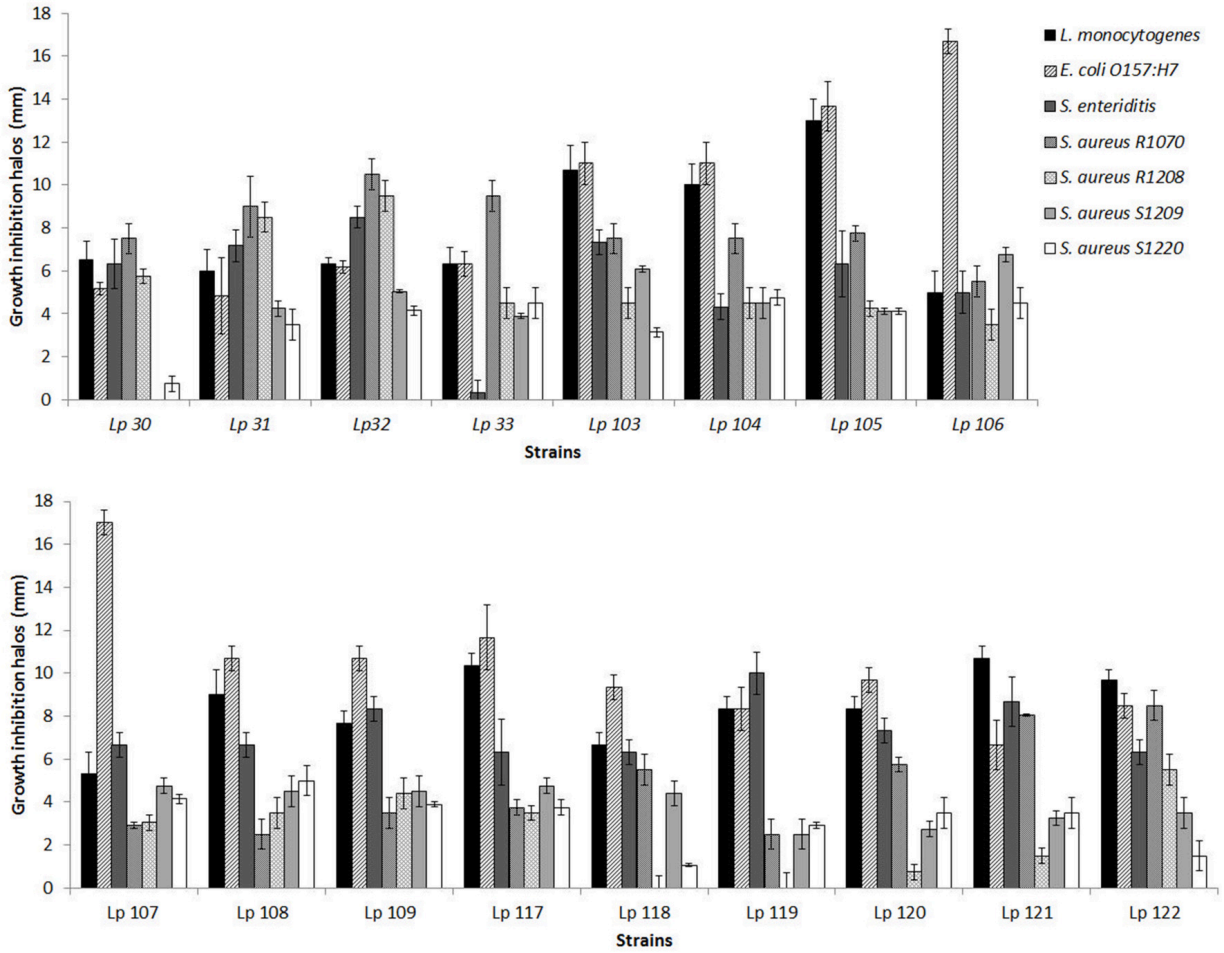
**Antimicrobial ability of selected ***L. plantarum*** strains against pathogenic bacteria as measured by agar spot assay**. Data are the mean ± SD of at least three independent experiments.

In this study, 79 *L. plantarum* strains were investigated for their possible antimicrobial activity against seven pathogenic bacteria, i.e., *L. monocytogenes, S. Enteritidis, E. coli* O157:H7, and four strains of *S. aureus*. The preliminary screening of all *Lactobacillus* strains, carried out by agar spot test, revealed a different range of antimicrobial activity, depending both on *L. plantarum* strain tested and on pathogen considered. Figure 1 reports data obtained with the best inhibiting *L. plantarum* strains, i.e. which determined overall inhibition halos of more than 5 radius mm, according to the classification proposed by Gaudana et al. (2010). As shown in Figure 1, some strains exhibited a very strong ability to inhibit the growth of food pathogens. In particular, *L. plantarum* 105 exhibited the major ability to inhibit *L. monocytogenes*, while both *L. plantarum* 106 and *L. plantarum* 107 presented the highest antagonistic effect on growth of *E. coli* O157:H7. *S. Enteritidis, S. aureus* R1070, R1208, S1209, and S1220 were mainly inhibited by *L. plantarum* 119, *L. plantarum* 32, *L. plantarum* 106, and *L. plantarum* 108, respectively. Contrariwise, *L. plantarum* 118 and 119 did not show any inhibition effects on the growth of *S. aureus* R1208. Similarly, *L. plantarum* 30 was not able to affect the development of *S. aureus* S1209.

### Well-diffusion assays

The antagonistic effect of those *L. plantarum* strains which exhibited appreciable antimicrobial activity, as determined by agar spot test (Figure 1), was further assessed by well diffusion assay using CSFs. In contrast to the results obtained with the agar spot assays, no CFS was able to contrast the growth of pathogenic bacteria (data not shown). In order to ascertain if the concentration of the inhibiting substance could be not adequate to sustain antagonistic action, the CFSs were concentrated by lyophilization, prior to their use in well diffusion tests. The 10x concentrated CFSs exhibited inhibition activities that were similar to that previously obtained by agar spot test (Table 2). The concentrated and differently treated CFSs were also tested, however, the inhibiting effect was lost after pH neutralization, while enzymatic and heat treatment had no impact on the inhibitory effect (data not shown).

**Table 2:** **Antimicrobial activity of CFS of ***L. plantarum*** strains determined by well-diffusion assay and expressed as the size of inhibition zones around the wells (mm)**.

	**CFS-A**	**CFS-B**	**CFS-A**	**CFS-B**	**CFS-A**	**CFS-B**	**CFS-A**	**CFS-B**	**CFS-A**	**CFS-B**	**CFS-A**	**CFS-B**	**CFS-A**	**CFS-B**
	***E. coli O157:H7***		***L. monocytogenes***		***S. Enteritidis***		***S. aureus R1070***		***S. aureus R1208***		***S. aureus S1209***		***S. aureus S1220***	
*L. plantarum* 30	5.0	4.4	5.0	4.9	6.0	5.5	7.0	6.5	5.0	5.0	0.0	0.0	0.5	0.0
*L. plantarum 31*	3.3	3.1	5.0	5.0	7.0	6.4	8.0	6.3	6.0	5.5	4.0	4.0	2.5	2.0
*L. plantarum 32*	4.1	4.0	5.5	5.0	7.7	6.9	8.8	6.4	8.0	7.8	4.0	3.8	3.0	2.8
*L. plantarum 33*	4.3	4.3	5.4	5.0	1.0	1.0	7.6	7.0	3.0	2.0	4.0	3.8	3.0	3.0
*L. plantarum 103*	6.0	6.0	8.0	8.0	6.8	6.3	7.0	6.3	3.5	2.0	5.5	4.0	3.0	3.0
*L. plantarum 104*	5.5	5.4	8.7	8.5	4.0	3.6	7.0	6.2	3.0	2.0	4.0	4.0	4.0	3.5
*L. plantarum 105*	7.2	7.1	10.2	10.0	5.2	5.0	6.5	6.0	3.1	2.0	4.0	4.0	4.0	3.5
*L. plantarum 106*	11.0	11.0	9.2	8.8	5.0	5.0	5.0	5.0	3.0	2.0	5.5	5.0	4.0	3.5
*L. plantarum 107*	10.8	1.5	9.3	9.0	5.1	5.0	3.0	2.1	3.0	2.5	4.5	4.0	4.0	4.0
*L. plantarum 108*	6.0	6.0	4.3	4.3	4.8	4.7	2.0	1.9	3.1	3.0	4.5	4.0	4.8	4.0
*L. plantarum 109*	6.3	6.0	4.3	4.5	7.0	7.2	3.0	2.5	4.0	3.2	4.5	4.5	4.0	3.6
*L. plantarum 117*	4.2	4.3	7.2	7.0	6.0	5.5	3.1	2.6	3.0	3.0	4.5	4.0	4.0	3.6
*L. plantarum 118*	4.6	4.5	5.5	5.3	5.9	5.8	5.0	4.9	1.0	1.0	4.0	4.0	1.0	1.0
*L. plantarum 119*	4.1	4.0	6.9	6.8	7.6	7.6	2.0	2.0	0.0	0.0	2.5	2.0	3.0	2.0
*L. plantarum 120*	3.9	3.8	6.8	6.8	8.0	7.9	5.0	4.9	0.0	0.0	2.5	2.0	3.0	2.5
*L. plantarum 121*	5.2	5.2	8.8	8.8	7.0	7.0	7.4	7.0	1.0	0.0	3.0	3.0	3.0	2.5
*L. plantarum 122*	6.9	6.5	8.9	8.8	5.4	5.0	7.1	7.0	4.0	4.0	3.0	3.0	1.0	1.0

*CFS-A, untreated and 10x concentrated cell free supernatant; CFS-B, 10x concentrated CFS heated at 80°C for 10 min*.

### Broth microdilution assays

**Figure 2:**
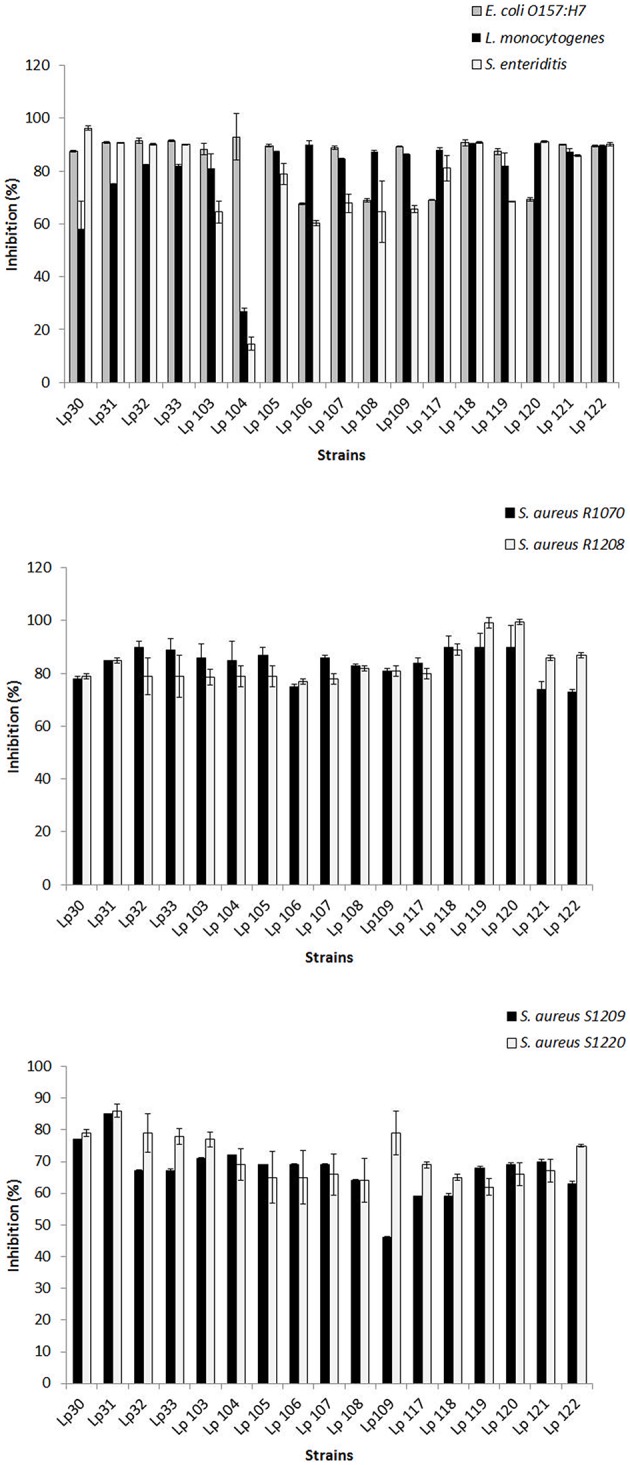
**Antimicrobial activity of CFSs-A (25%) of selected ***L. plantarum*** strains as determined by micro-dilution method**. Data are the mean ± SD of at least three independent experiments.

CFSs were further tested against pathogens by broth microdilution method. Interestingly, untreated CFSs from all selected *L. plantarum* strains determined significant inhibition of pathogen growth when used at ≥25% (25:75, CFS:growth medium), i.e., 30, 40, 50, 60, 70, 80, 90% (data not shown). Figure 2 shows the antagonism resulted by untreated CFSs of *Lactobacillus* strains using 25% of CFSs-A. *E. coli* O157:H7 growth was reduced by around 70 and 93% by CFS of *L. plantarum* 108 and 104, respectively. *L. monocytogenes* was inhibited up to 90% by *L. plantarum* 116, while the growth of *S. Enteritidis* was reduced by 96 % in presence of *L. plantarum* 30 CFS. The growth of all strains of *S. aureus* was significantly contrasted by all CFSs and, the highest reductions were around 90% (*S. aureus* R1070), 99% (*S. aureus* R1208), 85% (*S. aureus* S1209), and 86% (*S. aureus* S1220). Based on these findings, an aliquot consisting of 100 μl of untreated, 2-fold diluted CFSs was determined as the amount of CFSs showing more than 50% of inhibition ability for 98% of cases analyzed, and chosen for the further assays. The only two-fold diluted CFS causing an inhibition lower than 50% was that from *L. plantarum* 104 against *L. monocytogens* and *S. Enteritidis*.

In order to investigate on the nature of the inhibitory substances secreted by each *L. plantarum* strain showing antagonistic effects, CFS-As were submitted to different treatments. As a result, the potential inhibiting molecules lost their antagonistic ability after pH neutralization (data not shown).

### Statistical relationship among lactobacilli for their antimicrobial activity

**Figure 3:**
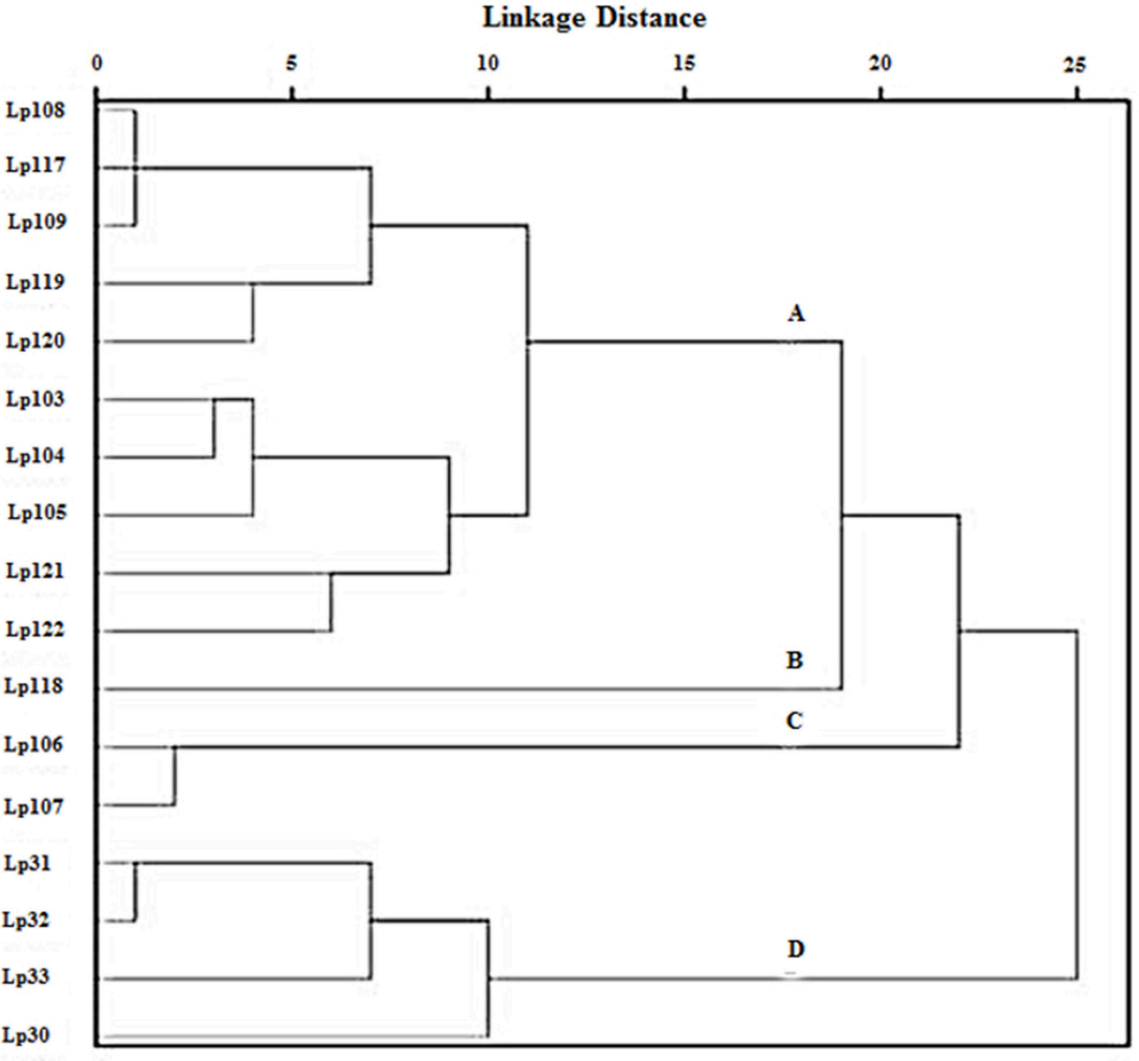
**Clustering of ***L. plantarum*** strains as a function of their antimicrobial activity against pathogens as observed by agar spot test**. Statistically significant difference (*p* < 0.005) among subgroups **(A), (B), (C),** and **(D)** was determined by ANOVA test.

Cluster analysis was performed on the inhibition halos data obtained by agar spot test, in order to group *Lactobacillus* strains showing similar antimicrobial activity. Euclidean distance was used to measure the proximity between two data and average linkage clustering was used as linkage criteria. As a result, four clusters of lactobacilli were distinguished (Figure 3). Group A contained 10 strains of *L. plantarum* (103, 104, 105, 108, 109, 117, 119, 120, 121, 122); group B was constituted of strain 118, group C consisted of *L. plantarum* 106 and 107; group D comprised four strains of *L. plantarum* (30, 31, 32, 33). Furthermore, ANOVA was elected as a method to study the statistical differences (*p* < 0.005) among the four groups (data not shown). Overall, the results indicated that the strains included in group A were able to contrast the growth of *L. monocytogenes* significantly better than strains in groups B, C, and D. *L. plantarum* 118, within group B, showed no or very low activity against *S. aureus* R1208 and *S. aureus* S1220, while *L. plantarum* 106 and *L. plantarum* 107, belonging to group C, were mostly active against *E. coli* O157:H7. Group D strains, including *L. plantarum* 30, 31, 32, and 33, were able to inhibit the growth of *S. aureus* R1208 significantly better than lactobacilli of group A, B, and C.

## Discussion

All analyzed *Lactobacillus* strains were shown to inhibit the growth of pathogens in a lactobacillus strain- and pathogen strain-depending manner. Using the agar spot method, 17 *L. plantarum* strains were identified as very strong inhibitors, according to the classification made by Gaudana et al. (2010), as they showed inhibition halos of more than 5 mm against the majority of the food pathogens tested. Cluster analysis was useful to group the *Lactobacillus* strains in four clusters, each of them denoted by a different antimicrobial activity. Within each group, peculiar abilities to contrast the growth of target pathogens were underlined such as a higher inhibitory activity against Gram-positive bacteria *L. monocytogenes* and *S. aureus* R1208 and Gram-negative bacteria *E. coli* O157:H7 by group A, group D, and group C, respectively.

The antimicrobial activity of the tested *L. plantarum* strains was mostly observed when they were grown on solid media and then brought into contact with pathogenic bacteria. This could be a considerable feature to be sought in the choice of starter or probiotic microorganisms. Indeed, live microorganisms carry out antimicrobial and preservative activity in the food when used as starters. Moreover, as probiotics, they can provide a protective benefit for the consumer when, following ingestion, can activate their metabolism in the intestine (De Vuyst and Leroy, 2007; Tejero-Sariñena et al., 2012; Arena et al., 2014).

The antimicrobial capability was also confirmed when 10x concentrated CFSs from *L. plantarum* strains were used in agar well diffusion assay, thus indicating that a minimal concentration of antimicrobial compounds is necessary to sustain that antagonism. CSF may include also other molecules, besides those effectively secreted by bacteria (i.e., medium components and/or intracellular compounds which may be accidentally released during CFS preparation). However CFS are routinely used to preliminarily screen the antimicrobial capacity of lactobacilli by well diffusion method (Herreros et al., 2005; Guo et al., 2010; Al Kassaa et al., 2014; Wang et al., 2014). For most of the tested *L. plantarum* strains, we found a good correspondence between the antimicrobial activities as assessed by either agar spot test or by well diffusion, using CFS. This indicates that the inhibitory effects mainly depend on exudates (which are included in the CFS) and only in part, if any, on other antagonistic mechanisms which require a more direct interaction, possibly occurring during co-culture on solid media (e.g., metabolic competition).

In addition, 1x CFSs were tested by broth microdilution method in order to understand whether the CFSs components could reduce the growth of pathogens in liquid-medium respect to agar-medium. The results suggested a greater capability of CFSs to contrast pathogenic bacteria in liquid-medium than in agar plates. Minimum amounts of CFS with inhibitory effect on pathogens were determined, indicating that two-fold diluted CFSs could exhibit more than 50% of inhibition for 98% of the cases analyzed. Absent or low activity by not concentrated CFSs in agar well diffusion tests was also previously reported (Saadatzadeh et al., 2013). It was also demonstrated that the antimicrobial effect was improved by CFS lyophililization and, more interestingly, such procedure was praised as an innovative strategy in bacterial products preparation (Saadatzadeh et al., 2013).

In order to investigate on the nature of the inhibitory substances secreted by each *L. plantarum* strain, both 1x CFSs and 10x CFSs were submitted to different treatments and tested against pathogens by broth method and well diffusion assay, respectively. Since pH neutralization eliminated the antimicrobial feature of CSF, while neither protease nor heat treatment had any impact on the inhibitory effect (both in sequential and in separate treatments, see the experimental section), we hypothesize that acidic pH and/or to the presence of organic acids could account for most of the observed antimicrobial activity. Indeed, although CFSs from bacterial cultures may contain many cellular metabolites, organic acids have been indicated as the principal antimicrobial agents when the antimicrobial activity is reduced or eliminated by alkaline neutralization (Bilkova et al., 2011; Zhang et al., 2011; Tejero-Sariñena et al., 2012). However, data obtained by CFS neutralization provide only a preliminary indication of the active compounds. Further experiments, including monitoring the pH of CFS and the acidification of growing cultures, as well as HPLC analysis, could help to better substantiate the potential role of organic acids. Moreover, organic acids, if any, could have enhanced the activity of other antimicrobial metabolites, which might require acidification and/or acid-mediated cell membrane disruption to exert an apparent antagonistic effect (Alakomi et al., 2000).

The antimicrobial effect of organic acids has been observed for several lactobacilli. The growth-inhibiting activity of different LAB, i.e., strains belonging to *Lactobacillus, Bifidobacterium, Lactococcus, Streptococcus*, and *Bacillus* genera, against pathogens such as *Salmonella typhimurium, E. coli, Enterococcus faecalis, S. aureus*, and *Clostridium difficile*, was attributed to a pH reduction and/or to the production of organic acids, including lactate and acetic acid (Tejero-Sariñena et al., 2012). Moreover, De Keersmaecker et al. (2006) also found a strong antimicrobial activity of *L. rhamnosus* strains against *S. typhimurium*, which was ascribed to the accumulation of lactic acid.

The organic acids secreted by LAB determine an environmental pH reduction that can be adverse for those microorganisms sharing the same niche (Tharmaraj and Shah, 2009). In their undissociated form, organic acids can penetrate the cytoplasmic membrane of target microorganisms, thus resulting in intracellular acidification and in the collapse of the transmembrane proton motive force. Such mode of action is pH-dependent, because the undissociated forms are prevalent when the pH value is below the pKa of the organic acid (Batish et al., 1997; Dalié et al., 2010; Schillinger and Villareal, 2010; Cortés-Zavaleta et al., 2014). Accordingly, the production of undissociated organic acids was indicated as the main mechanism through which several intestinal lactobacilli contrast the growth of a range of both Gram-negative and Gram-positive pathogenic bacteria in liquid media (Annuk et al., 2003; Topisirovic et al., 2007; Toy et al., 2015). Despite, the neutral pH conditions of the large intestine, probiotic bacteria could produce locally high concentrations of organic acids, thus establishing chemical microenvironments where the antagonistic action can be carried out (Alakomi et al., 2000).

Besides their pH lowering properties, sometimes, the antimicrobial effect of organic acids reflects a specific mode of action which may be relatively pH-independent. For instance, lactate, i.e., the main acid produced by LAB fermentation, was proved to specifically permeabilize the outer membrane of Gram negative species, causing structural alterations in the phospholipid component (Alakomi et al., 2000). Likewise, probiotic lactobacilli inhibited *Campylobacter jejuni* growth by the secretion of lactic acid, which was shown to disrupt membrane integrity through a mechanism which is not solely pH-dependent (Neal-McKinney et al., 2012).

In the last decades, the main molecules which have been extensively studied as antimicrobial agents have been bacteriocins (Adebayo et al., 2014; Gupta and Srivastava, 2014). Bacteriocins are ribosomally-synthesized peptides that can act against bacteria of the same species (narrow spectrum) or of the same genera (broad spectrum). These compounds can be produced directly in fermented food either by bacteriocin-producing starter cultures (fermentative and bioconservative actions) or by protective culture strains (only bioconservative action). Additionally, isolated and purified bacteriocins can be used as food additives or included in the packaging materials (Kos et al., 2011; Fan and Song, 2013). One of the major drawbacks in the use of bacteriocins as natural antimicrobials is that their proteinaceous structure can be easily altered by diverse proteases possibly occurring in the food, being secreted by different bacteria and/or occurring in the human digestive tract (Saavedra et al., 2004). Moreover, the efficacy of bacteriocins in food can be decreased by their adsorption to food components, poor solubility and uneven distribution within the food matrix (Hartmann et al., 2011). Compared to LAB bacteriocins, which are mainly active against Gram-positive bacteria, organic acids exhibit a broader spectrum of antimicrobial action. Besides, organic acids are not sensitive to proteases and may be better solubilized. Therefore, the bioprotective potential of organic acid-producing LAB is high and suited to wide applications in food safety and nutritional medicine (Mu et al., 2012; Pawlowska et al., 2012).

The use of CFS as antimicrobial ingredients could be an interesting strategy in food preparation. CFS produced by selected bacteria could be effective in inhibiting pathogens, especially when the inoculation of live inhibiting microorganisms may not be feasible, e.g., in food subjected to refrigeration in which the psychrotrophic *L. monocytogenes*, but not lactobacilli, could easily grow. Furthermore, the use of CFS rather than purified antimicrobials could determine the advantage to have different biologically active substances, with possible synergistic effects, in one product (Hartmann et al., 2011). It is worthwhile mentioning that some recent *in vitro* studies have suggested potential and intriguing biomedical applications of CFSs. For instance, CFS from LAB, with antimicrobial activity against *C. difficile*, was proposed as a plausible alternative to the therapies for the treatment of CD-associated gut disorders (Joong-Su et al., 2013). Moreover, CFSs from *Lactobacillus* strains were ascribed health beneficial effects, including inhibition of cancer metastatis (Escamilla et al., 2012), positive modulation of the intestinal immune response (Bermudez-Brito et al., 2013) and cholesterol-reducing properties (Kim et al., 2008).

To sum up, this study provides evidence that several of the screened *L. plantarum* strains possess a significant ability to contrast various pathogenic bacteria, including both Gram negative and Gram positive species, which can contaminate food and are responsible for diseases in humans. The biosynthesis of organic acids is proposed as one of the main mechanism through which the antimicrobial activity is exerted. The antagonistic feature could be a distinctive trait to take into account for the selection of starters and or probiotics that could also function as bio-control agents against potentially harmful microorganisms during food processing and storage. In a future perspective of reducing or eliminating the use of chemical compounds, this work contributes to the existing knowledge in a context in which the consumer's attention is increasingly aimed at healthy and natural food products.

## Author contributions

GS designed the experimental plan, analyzed the results and read the final paper. MA made the experimental trials and wrote up the paper. AS supported the technical part. GN provided some biological materials, participated to the experimental plan and read the final paper. FG and DD participated to the experimental plan and read the final paper. DF made the experimental trials and read the final paper.

### Conflict of interest statement

The authors declare that the research was conducted in the absence of any commercial or financial relationships that could be construed as a potential conflict of interest.
